# *Bordetella pertussis*: An agent not to be forgotten in Qatar

**DOI:** 10.5339/qmj.2021.10

**Published:** 2021-03-12

**Authors:** Samina Hasnain, Jesha Mundodan, Soha Al Bayat, Hayat Khogali, Hamad Al-Romaihi

**Affiliations:** EPI Section, Health Promotion and Communicable Disease Control, Ministry of Public Health, Doha, Qatar jmundodan@moph.gov.qa

**Keywords:** Pertussis, *Bordetella pertussis*, Qatar, vaccine preventable disease, re-emergence, maternal immunization

## Abstract

Background: Pertussis (whooping cough) is a vaccine-preventable disease caused by the bacterium *Bordetella pertussis* that is spread by airborne respiratory droplets. Clinical symptoms vary from infants to adults and are most contagious before the onset of symptoms. Infants are at the highest risk of infection, especially before they are old enough to receive at least two doses of pertussis-containing vaccine. There have been no indigenous cases of pertussis in Qatar since 2010 until 2018, due to free pertussis-containing vaccines under the National Immunization Schedule of Qatar, with coverage consistently above 95%. Two cases were reported in 2016 but were found to be imported. In 2019, 20 infants were reported as suspected pertussis to the Health Promotion and Communicable Disease Control (HP-CDC), Ministry of Public Health (MOPH), Qatar; of them, five were laboratory confirmed as pertussis.

Objective: This study aimed to describe the five confirmed cases of pertussis reported to HP-CDC, MOPH, Qatar, between January 1 and December 30, 2019.

Summary of Cases: All five confirmed pertussis cases were under one year old, and three were boys. All except one were immunized-for-age, and three had not received any doses of pertussis-containing vaccine and in none of the cases had the mother received tetanus, diphtheria, and acellular pertussis (Tdap) vaccine during pregnancy. All infants were born in Qatar, and two were Qatari nationals.

Conclusion: There may be a possibility of re-emergence of pertussis in Qatar. Active immunization and coverage maintenance are the best tools to prevent re-emergence. Undiagnosed and untreated pertussis cases are potential sources of infection. The partial or unimmunized groups may be significantly at risk, especially during infancy and before reaching the age to complete the three primary doses of diphtheria, tetanus, and pertussis vaccines. Focus on increasing awareness among those providing antenatal care, regarding the importance of Tdap vaccination during pregnancy, is necessary.

## Disease Overview

Pertussis is an acute respiratory disease caused by *Bordetella pertussis*, a gram-negative bacterial pathogen, which spread by airborne respiratory droplets, with an incubation period of 7–20 days. Clinical symptoms vary from infants to adults, which may include paroxysms of cough followed by a high-pitched inspiratory whoop. The whoop is less likely to be heard in infants and adults. Infants may develop apnea, cyanosis, and postcoughing fits and may also have associated posttussive vomiting. Adults generally report irritating cough that can last for weeks. Coughing fits due to pertussis infection can last for up to 10 weeks or more. Nasopharyngeal swab culture or rapid tests may be used for diagnosis. Clinicians generally treat pertussis with antibiotics, which are used to control symptoms and prevent infected people from spreading the disease.^1^

Pertussis can cause serious illness in people of all ages and can even be life threatening. Hospitalization may be required particularly in infants less than six months and in severe cases with complications such as apnea, cyanosis, pneumonia, seizures, or encephalopathy. The risk of infection and severity of morbidity is maximum at an age before infants are old enough to receive at least two doses of pertussis-containing vaccine.^1^ Children between 11 and 18 years are also at risk, as the immunity starts waning.^2^

Globally, it is estimated that there are 24.1 million pertussis cases and 160,700 deaths from pertussis in children  < 5 years of age according to recent modeling data, with periodic epidemics occurring every 2–5 years.^3^ Data from the United States revealed that 4000 cases were reported annually in the 1980s, and it increased to 48,277 in 2012.^4^ In 2016, 17,972 cases were reported, with an incidence rate of 70.9 per 100,000 in children  < 6 months, 31.9 per 100,000 in 6–11 months old, and 13.7, 14.8, and 16.3 per 100,000 for those aged 1–6, 7–10, and 11–19 years, respectively.^5^ However, the underreporting of mild cases would lower these rates. In Europe, 48,446 pertussis cases were reported to the European Surveillance System by 30 European Union/European Economic Area countries in 2016.^6^ This number was slightly higher than that reported in 2012 (42,572 cases), the year of peak pertussis incidence in Europe. In the USA, rates were higher among children less than one year old. In this younger group, the notification rate was 73.6 cases per 100,000 population, a value significantly higher than that reported in 2014 (51.6 per 100,000 population). Moreover, children between 10 and 14 years old had the second highest incidence rates of pertussis, approximately 30 cases per 100,000 population.^7^

The most effective way to prevent pertussis is through vaccination using pertussis-containing vaccines.^8^ Vaccinated children and adults can become infected with and spread pertussis; however, disease is typically much less serious in vaccinated people. Sporadic outbreaks of pertussis have been noted in many parts of the world including countries with high vaccination coverage.^9,10^ Brazil started the systematic control of pertussis in 1983, with the inclusion of diphtheria, tetanus, and pertussis (DTP) vaccine in children's basic vaccine schedule, around the time when developed countries indicated the first signs of disease resurgence.^11^ In 1990, vaccination coverage increased to approximately 70%, and the incidence of the disease decreased from 30 to 10.6 per 100,000 inhabitants. In the last decade, the incidence of pertussis in Brazil remained stable, ranging from 0.72 to 0.32 per 100,000 inhabitants in 2004 and 2010, respectively. In 2011, there was a sudden increase in the number of confirmed cases in relation to the previous 5 years, increasing the incidence to 1.2 per 100,000 inhabitants, even though high vaccination coverage was maintained.^12^

**Figure 1.**
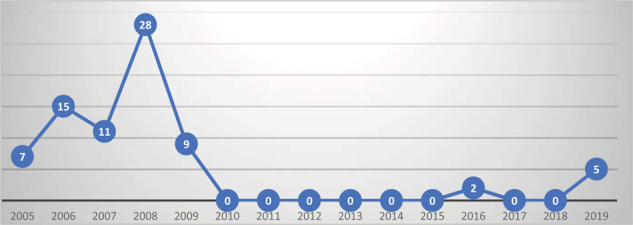
Pertussis cases reported in Qatar between 2005 and 2019

Figure 1 shows the time trend of pertussis cases in Qatar from 2005 to 2019. There have been no indigenous cases of pertussis in Qatar since 2010 until 2018. Two cases were reported in 2016, but on investigation, both of these cases had a history of recent travel to endemic countries and, therefore, were classified as imported cases. In 2019, 20 cases were reported as suspected pertussis to the Health Promotion and Communicable Disease Control (HP-CDC), Ministry of Public Health (MOPH), Qatar. All 20 underwent tests using polymerase chain reaction (PCR), and 5 cases were laboratory confirmed as pertussis.^13^

The following is the summary of the five confirmed cases of pertussis reported to HP-CDC, MOPH, Qatar, between January 1 and December 30, 2019 (Table 1).

Case 1 is a two-month-old American male patient who was admitted to a tertiary care hospital with a prolonged history of cough for one-month and recurrent fever. The child had repeated admissions to the pediatric emergency center (PEC) with bronchiolitis. He presented with classic whooping cough on admission and was started on antibiotics, after which his cough started to improve. His throat swab was positive for pertussis and respiratory syncytial virus on PCR test. He was born in Qatar as a full-term baby, and the mother had not received tetanus, diphtheria, and acellular pertussis (Tdap) vaccination during pregnancy. He had received bacillus Calmette–Guérin (BCG) and hepatitis B vaccines at birth but missed the two-month immunization as he was ill. He had no history of travel outside the country. His grandparents from the USA visited them, and both had tested negative for pertussis. His two elder siblings aged 10 and 17 years also had a history of prolonged cough for 1 month, but both tested negative for pertussis. The mother also informed that there were several children in their neighborhood with similar cough.

Case 2 is a six-week-old Pakistani female patient, who had previously presented to PEC with a runny nose and cough with breath-holding spells, was admitted in the pediatric intensive care unit (PICU) due to worsening of symptoms, with the chief complaints of persistent cough, runny nose, and breath-holding spells with bluish discoloration of the face suggestive of cyanosis for 15 days. Her throat swab tested positive for *B. pertussis* using PCR. The child was started on antibiotics and improved. She was a full-term baby delivered via vacuum extraction in Qatar and had received BCG and hepatitis B vaccines at birth; she was not due for any further vaccines at the time of developing symptoms. The mother had not received Tdap vaccination during pregnancy. She had no other siblings and was living with parents and grandparents. The grandmother also reported a history of cough of three weeks but was not tested for pertussis.

Case 3 is an eight-month-old Qatari female patient, who presented to PEC with a three-day history of cough followed by a whoop, difficulty breathing, fever, and vomiting with poor oral intake. On examination, the child was in respiratory distress with tachypnea, chest retractions, and bilaterally decreased breath sounds with wheeze. The child was transferred to the PICU and treated with antibiotics, bronchodilators, and steroids. She was initially given oxygen by nasal cannula but was later escalated to bilevel positive airway pressure (BiPAP) and continuous positive airway pressure (CPAP) on worsening. Chest radiography showed bilateral infiltrates with hyperinflation. PCR test was positive for *B. pertussis*, mycoplasma, rhinovirus, and parainfluenza viruses. The blood cultures were negative. The child was discharged home after two weeks against medical advice, on cessation of BiPAP and CPAP. Two elder siblings, who had traveled to Germany with their father a month back, had coughs, but they did not require hospitalization and were not tested because of parental refusal. The mother reported that a three-month-old cousin, who she believes was vaccinated for age, had a similar condition but did not require hospitalization. The child was born in Qatar, and the mother had not received Tdap vaccination during pregnancy. The child had not received any further vaccines after BCG and hepatitis B vaccines at birth, as the family is vaccine hesitant, but the six elder siblings have been vaccinated for age. The child was lost to follow up on investigating about the vaccine hesitancy in the family.

Case 4 is that of a six-month-old Sudanese male patient known to have glucose-6-phosphate dehydrogenase deficiency, who repeatedly presented to the PEC with a history of paroxysmal cough episodes for two days, occasionally ending with breath holding, wheezing, and face redness. The child was feeding well. Chest radiography showed no abnormality. The child was referred to the tertiary hospital as a case of pertussis-like syndrome where he was diagnosed to have pertussis based on a positive PCR result using a throat swab sample. The child was born in Qatar and had been vaccinated for his age except the six-month immunization due to the current illness. The mother had not received Tdap vaccination during pregnancy. The child did not have any travel history outside the country before the onset of symptoms or any significant family history of similar illness.

Case 5 is a two-month-old Qatari male patient, who presented with a history of cough for one week and difficulty in breathing for two weeks. There was no history of fever or feeding difficulty. On worsening of symptoms, the child was referred to the tertiary hospital where throat swab was taken and diagnosed to be positive for *B. pertussis* and influenza A by PCR. The child was started on antibiotics and kept under observation overnight in the hospital and discharged the next day on parent's request. The child was born in Qatar and had received BCG and hepatitis B vaccines at birth and one dose of pertussis-containing vaccine. The mother had not received Tdap vaccination during pregnancy. The child had no travel history outside the country. The two elder siblings are fully immunized for their age, and one sibling had mild cough. The mother also reported a history of contact with his teenage aunt who also had recent cough and cold symptoms.

## Discussion

Qatar has an extremely mobile expatriate population, which poses a significant challenge to the control of communicable diseases due to the temporary variation in vaccine coverage and importation of different strains of infectious agents. Undiagnosed and untreated pertussis cases are potential sources of infection transmission. Moreover, the infection is most contagious before onset of symptoms.^1^

Pertussis-like syndrome, a clinical picture such as whooping cough with recurrent persistent or paroxysmal coughing followed by a whoop or vomiting, is a common lower respiratory syndrome seen in pediatric practice. Commonly identified infections in children presenting with pertussis-like syndrome in order of frequency include respiratory syncytial virus, *B. pertussis,* adenovirus, influenza A, and human metapneumovirus. Coinfections are also common. Pertussis-like syndrome can occur at all ages but is more common in children. Often, children presenting to emergency departments or pediatric clinics with a history of prolonged spasmodic cough or posttussive whooping and vomiting were reported as having pertussis-like syndrome without a conclusive diagnosis and treated presumptively. The clinical manifestations of these viral infections are indistinguishable from pertussis; therefore, the diagnosis of pertussis infection cannot be conducted by clinical symptoms only and should be confirmed by laboratory tests.^14–16^

### Better diagnostic methods

It could be speculated that resurgence of pertussis may be due to the availability of newer and more rapid diagnostic tests. Previously, the only diagnostic method available was nasopharyngeal swab cultures to confirm *B. pertussis* infection, which had 100% specificity but low sensitivity, ranging from 12% to 60%.^17^ The nasopharyngeal samples for culture had to be collected within the first 15 days of the disease. At this stage, the symptoms may be nonspecific and difficult to diagnose, especially in children above one year of age. Isolation from culture is also difficult if the patient has recently been treated with antibiotics before swab collection.^17^ Molecular techniques, such as real-time (RT)-PCR, have now become widely available, allowing rapid diagnosis of pertussis. These tests are also more sensitive (70%–99%) as they do not require viable bacteria.^17,18^ Serologic tests to detect antipertussis toxin immunoglobulin G in serum and saliva have also been developed. Several countries such as Canada and Australia have reported a marked increase in pertussis cases, soon after reliable and rapid tests for pertussis diagnosis were available.^17,18^

In Qatar, up until 2014, most cases were identified based on clinical diagnosis alone as blood cultures often yielded negative results. Diagnostic tests such as PCR were introduced in 2014.

### Underreporting of cases

Pertussis cases may be underreported due to low disease awareness, lack of surveillance data and reliable reporting systems, absence of clear case definition, especially for cases above one year of age, and limited laboratory diagnostics.^18^ Case definitions for pertussis in different age groups have recently been revised to improve the epidemiological surveillance of the disease.

### Infant immunization schedule and coverage

The availability of pertussis vaccines in the child immunization schedule has reduced pertussis incidence globally with decreased hospitalization rates and deaths. Widespread use of the whole-cell pertussis vaccine (wPV) series in infants led to a marked reduction in pertussis cases, hospitalization rates, and deaths. However, wPV can cause local and systemic adverse events, which has led to vaccine hesitancy among many parents and healthcare providers and increased demand for producing acellular pertussis vaccine (aPV). The aPV is less reactive and has a better safety profile, but it is more expensive to produce; therefore, it was initially used in wealthier industrialized countries, whereas low-middle income countries continued the use of wPV.^17^

**Figure 2.**
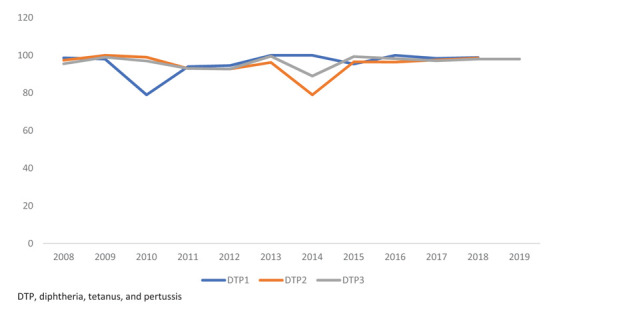
DTP vaccination coverage in Qatar, 2008-2019

In Qatar, pertussis-containing vaccines are free under the National Immunization Schedule for infants and children, which included five doses of wPV before 2010. With the availability of the hexavalent vaccines (diphtheria, tetanus, and pertussis [DTaP], hemophilus influenza type B, Hepatitis B, and inactivated polio vaccines), the number of wPV doses was reduced to two doses with one dose of aPV till 2015. In the current schedule, 2 doses of aPV are given during infancy at 2 and 4 months with 1 dose of wPV at 6 months of age, followed by 3 booster doses at the age of 18 months, 4–6 years, and 13–16 years. The vaccination coverage for the third dose of DTP has been consistently maintained above 95% since a long time; however, a dip in coverage was noticed in 2010 when hexavalent was first introduced into the schedule (Figure 2). Another dip in coverage occurred in 2014, when the Cerner electronic medical recording system was introduced in the primary healthcare corporation, requiring well-baby clinics to shift from a walk-in vaccination to an appointment-based service, which allowed a maximum of 25 children to be vaccinated per shift.^13^ For the booster dose at 13–16 years, the vaccination coverage lingers between 50% and 70%. Since 2011, the MOPH has been conducting nationwide Tdap campaigns for grade 10 students to boost vaccine coverage.

### Waning of immunity

Although neither natural infection nor immunization with wPV provides long-term immunity, increasing pertussis incidence was observed in countries that had switched from wPV to aPV, suggesting a more rapid waning of immunity after immunization with aPV. The World Health Organization (WHO), therefore, recommends that at least one dose of wPV for priming and three to four doses of aPV should be used for boosting all infant immunization programs to prevent rapid waning of immunity.^17–19^ Shankar reported a case of infection in a child with completed primary immunization.^20^ It appears that pertussis incidence has increased in school-age children in North America and western Europe, where aPV is used, but an increase has also occurred in some countries that use wPV.^3^

### Genetic modifications of *B. pertussis*

Circulation of *B. pertussis* strains with modified or absent antigens may also reduce the efficacy of aPV and contribute to the more rapid waning of immune response. This includes the recent emergence of *B. pertussis* and parapertussis strains with the loss of pertactin (PRN) production, which is an important virulence factor included in aPV.^21–24^ However, a US-based study assessed the vaccine effectiveness (VE) of a five-dose DTaP series among 4–10 year-olds and a Tdap booster among 11–19 year-olds in an area where over 90% of *B. pertussis* strains were PRN deficient. There were no significant differences in VE for DTaP (84%; 95% confidence interval [CI] 58–94) and Tdap (70%; 95% CI 54–81) in areas with predominant circulation of PRN-positive strains. Another US-based study showed that the severity of pertussis infections is worse if the infecting strain was PRN-negative compared with PRN-positive.^23,24^ Therefore, theoretically, aPV use may increase the emergence of mutated *B. pertussis* strains, but the effect of this phenomenon on vaccines efficacy and VE needs to be fully evaluated.

### Maternal immunization

Pertussis infection during early infancy can have the most devastating consequences, including hospitalization, undernutrition, respiratory distress, and death. Since newborns below six weeks of age cannot receive pertussis vaccination, the CDC and WHO recommend that the best way to provide protection is to vaccinate all expectant mothers during the last trimester of pregnancy. Vaccination of pregnant women with Tdap is especially important to help protect babies before they reach the age to receive at least three doses of pertussis-containing-vaccines. In addition, all caregivers of newborns and infants should receive pertussis vaccines at least one month before planned infant contact.^18,19,25^ The MOPH has published guidelines for maternal vaccination with Tdap to be given in the last trimester of pregnancy in 2012. Although the exact estimate of the vaccine coverage in pregnant women in Qatar is not available, it is likely to be very low, especially due to vaccine hesitancy and lack of recommendation by obstetricians during antenatal care.

## Conclusion

Cough is a common complaint among children, and its causes are multiple. There may be a possibility of re-emergence of pertussis in Qatar. Undiagnosed and untreated pertussis cases are potential sources of infection. Therefore, increasing awareness among health professionals to the early suspicion of this illness, early diagnosis and management, and timely reporting are very crucial. The use of better diagnostic methods, for example, molecular techniques such as RT-PCR and serologic tests, may be one of the reasons for the apparent resurgence, which cannot be easily ruled out. The partially or unimmunized groups may be significantly at risk especially during infancy before reaching the age to complete the three primary doses of pertussis-containing vaccine.

### Recommendations

Active immunization and coverage maintenance are the best tools to prevent re-emergence. There is an increasing need to be exact on the timing of the fourth and sixth month vaccination. Focus on increasing awareness among those providing antenatal care, regarding the importance of Tdap vaccination during pregnancy, is necessary. The families and pregnant women should be educated for increased awareness of pertussis and vaccination, since it is vital for immunization against pertussis and decreases the cases. We must do intensive contact tracing and screen possible close contacts to curb the resurgence in Qatar.

## Ethical Considerations

This manuscript was approved by the Institutional Research Board of PHCC (PHCCDCR202011137).

## Conflicts Of Interest

The authors have no financial or other interests to declare.

## Figures and Tables

**Table 1:** Summary of confirmed Pertussis cases in Qatar between January 1 and December 30, 2019.

Case no	Patient age, nationality, gender	Presenting symptoms	Vaccination status	Place of birth & travel history	Investigations & treatment	Significant family history	Maternal vaccination with Tdap during pregnancy

1	Two months, American, male	Prolonged fever and cough for one month	BCG and hepatitis B at birth	Born in Qatar; no travel history	Chest X-ray: normal Throat swab: +ve PCR for pertussis and RSV Blood c/s: –ve treated with antibiotics	Two siblings—also had h/o cough. Both were vaccinated for age.	None

2	Six weeks, Pakistani, female	Persistent cough, runny nose, and breath-holding spells with bluish discoloration of the face for two weeks	BCG and hepatitis B at birth	Born in Qatar, no travel history	Chest X-ray: normal Throat swab: +ve PCR for pertussis Blood c/s: –ve treated with antibiotics	No siblings. Living with parents and grandparents. Grandparents had a h/o cough.	None

3	Eight months, Qatari, female	Cough followed by a whoop, difficulty breathing, fever, and vomiting with poor oral intake for three days Signs of respiratory distress with tachypnea, chest retractions, and bilaterally decreased breath sounds with wheeze	BCG and hepatitis B at birth	Born in Qatar, no travel history	Chest X-ray bilateral infiltrates with hyperinflation Throat swab: PCR test +ve for *Bordetella pertussis*, Mycoplasma, Rhinovirus, and Parainfluenza viruses Blood c/s : –ve treated with antibiotics and respiratory support (O2 and BiPaP/CPAP)	Six siblings—vaccinated for age. Two siblings and father had a recent h/o travel to Germany and reported having prolonged cough.	None

4	Six months, Sudanese, male	Paroxysmal cough ending with breath holding, wheeze, and redness of face for two days	BCG and hepatitis B at birth Hexavalent (DTaP, Hib, IPV, and hepatitis B), PCV 13, and Rotavirus vaccine at two and four months	Born in Qatar, no travel history	Chest X-ray: normal Throat swab: +ve PCR for pertussis Blood c/s: –ve treated with antibiotics	None	None

5	Two months, Qatari, male	Cough for two weeks, difficulty in breathing for one week	BCG and hepatitis B at birth Hexavalent, PCV 13, and Rotavirus vaccine at two months	Born in Qatar, no travel history	Chest X-ray: Normal Throat swab: *Bordetella pertussis* and influenza A by PCR Blood c/s: –ve	Two siblings-one had h/o cough. Both were vaccinated for age. Teenage aunt also had a h/o cough and cold-vaccination status unknown.	None


Tdap, tetanus, diphtheria, and acellular pertussis; BCG, bacillus Calmette–Guérin; PCR, polymerase chain reaction; RSV, respiratory syncytial virus; c/s, culture and sensitivity; h/o, history of; BiPAP, bilevel positive airway pressure; CPAP, continuous positive airway pressure; PCV 13, pneumococcal conjugate vaccine; Hib, hemophilus influenza B; DTaP, diphtheria, tetanus, and pertussis.
